# QRS-Corrected Prediction of the Diastolic Rest Period for Coronary CT Angiography in Patients with Complete Left Bundle Branch Block

**DOI:** 10.3390/jcdd13060285

**Published:** 2026-06-22

**Authors:** Tsubasa Morioka, Shingo Kato, Kouta Mitsutake, Hidenao Yanagisawa, Toshiharu Izumi, Tomokazu Sakano, Eiji Ishikawa, Hiroyuki Kamide, Daisuke Utsunomiya

**Affiliations:** 1 Department of Radiology, Yokohama City University Hospital, Yokohama 236-0004, Japan; morioka@yokohama-cu.ac.jp (T.M.); mitsutake.kot.hj@yokohama-cu.ac.jp (K.M.); yanagisawa.hid.bj@yokohama-cu.ac.jp (H.Y.); izumi3@yokohama-cu.ac.jp (T.I.); sakanotm@yokohama-cu.ac.jp (T.S.); cpa10233@yokohama-cu.ac.jp (E.I.); 2 Department of Diagnostic Radiology, Yokohama City University Graduate School of Medicine, Yokohama 236-0004, Japan; d_utsuno@yokohama-cu.ac.jp; 3 Department of Diagnostic Radiology, Yokohama City University Medical Center, Yokohama 232-0024, Japan; kamide.rad@gmail.com

**Keywords:** coronary CT angiography, cardiac phase, slow filling, bundle branch block, left bundle branch block, QRS duration

## Abstract

Background: Optimal phase selection in coronary computed tomography angiography (CCTA) is crucial. While the mid-diastolic slow-filling (SF) phase is typically predicted using a conventional formula based on heart rate and atrioventricular conduction time, its validity in complete left bundle branch block (CLBBB)—where pronounced QRS prolongation induces severe mechanical dyssynchrony—remains unclear. We evaluated the impact of bundle branch block on cardiac-phase selection and validated a QRS-corrected predictive model. Methods: We retrospectively analyzed 94 patients (sinus rhythm, *n* = 40; complete right bundle branch block [CRBBB], *n* = 36; CLBBB, *n* = 18). Measured SF at the proximal right coronary artery was compared against predictions from the conventional formula (SF = −362 + 0.742 × [RR − PQ]) and a proposed QRS-corrected formula incorporating a “−(QRS − 100)” subtraction. To test the necessity of a novel model, regression analyses were reconstructed exclusively for the CLBBB cohort. Results: In CLBBB patients, the conventional formula critically overestimated SF by an average of 37.9 ms (RMSE 42.5 ms). Reconstructing simple and multivariate regression models exclusively for the CLBBB group yielded coefficients remarkably similar to the conventional formula, indicating that the fundamental physiological dependency on RR and PQ intervals remains intact despite the bundle branch block. Crucially, the simple proposed QRS-corrected formula successfully eliminated the overestimation bias (mean error −6.9 ms; *p* = 0.176) and demonstrated the highest predictive accuracy (RMSE 21.2 ms). Conclusions: A completely new predictive regression model is unnecessary for CLBBB patients. Simply incorporating a theoretical subtraction of pathological QRS prolongation optimally corrects the diastolic resting phase.

## 1. Introduction

Coronary computed tomography angiography (CCTA) has become an established noninvasive imaging modality for the assessment of coronary artery disease. Recent advances in CT technology have improved spatial and temporal resolution, enabling more accurate visualization of the coronary arteries [1,2]. Nevertheless, coronary arteries remain subject to continuous motion throughout the cardiac cycle, and the selection of an appropriate reconstruction phase is essential for obtaining high-quality diagnostic images [3].

In patients with sinus rhythm and relatively low heart rates, the mid-diastolic slow-filling (SF) period is generally regarded as the most motion-free phase of the cardiac cycle and is commonly used for image reconstruction [4,5]. Previous studies have demonstrated that the duration of this diastolic coronary rest period can be estimated using predictive formulas based on the RR interval and atrioventricular conduction time (PQ interval) [6,7]. These formulas have been shown to provide reasonable estimates in patients with normal intraventricular conduction.

Complete right bundle branch block (CRBBB) and complete left bundle branch block (CLBBB) are characterized by prolonged QRS duration and altered ventricular activation patterns, resulting in electrical dyssynchrony [8,9]. Electrical dyssynchrony may subsequently lead to mechanical dyssynchrony and altered ventricular contraction–relaxation timing [10,11,12]. Mechanical dyssynchrony has been shown to result in abnormal regional myocardial deformation and altered ventricular performance [13,14,15,16].

Left bundle branch block (CLBBB), in particular, is associated with delayed activation of the left ventricular free wall and abnormal septal motion, resulting in impaired ventricular synchrony [17,18,19]. These abnormalities have been linked to altered regional myocardial mechanics and impaired ventricular coordination [17,20,21]. In contrast, the physiological impact of CRBBB on left ventricular mechanics is generally considered less pronounced [22,23].

Because coronary artery motion is closely linked to ventricular contraction and relaxation, abnormalities in ventricular activation may affect the timing and duration of coronary motion-free phases. Prior studies have demonstrated that mechanical dyssynchrony can influence myocardial work distribution and ventricular timing, potentially affecting global cardiac mechanics [24,25]. However, despite the widespread use of CCTA, relatively little is known about how bundle branch block influences coronary resting phases during the cardiac cycle. Furthermore, it remains unclear whether conventional diastolic prediction formulas derived from patients with normal conduction remain applicable in patients with bundle branch block.

The present study was designed to investigate the impact of bundle branch block on coronary motion-free phases during CCTA. Specifically, we quantitatively compared diastolic slow-filling duration and systolic resting phase characteristics among patients with sinus rhythm, CRBBB, and CLBBB. In addition, we evaluated the performance of the conventional SF prediction formula and examined whether a QRS-adjusted correction could improve prediction of the diastolic resting period in patients with CLBBB.

## 2. Materials and Methods

### 2.1. Study Design and Population

This was a single-center retrospective observational study. Among patients who underwent coronary computed tomography angiography (CCTA) at our institution, those presenting with sinus rhythm, sinus rhythm with complete right bundle branch block (CRBBB), or sinus rhythm with complete left bundle branch block (CLBBB) on electrocardiography were included. A total of 94 patients were analyzed: 40 in the sinus rhythm group, 36 in the CRBBB group, and 18 in the CLBBB group.

Patients with irregular RR intervals, such as atrial fibrillation or advanced atrioventricular block, and those in whom image analysis was difficult due to severe motion artifacts were excluded. The study protocol was approved by the institutional ethics committee. Because of the retrospective nature of the study, the requirement for informed consent was waived, and an opt-out approach was used.

### 2.2. CT Acquisition Protocol

CCTA was performed using a 320-row detector CT scanner (Aquilion ONE PRISM Edition; Canon Medical Systems Corporation, Otawara, Japan).

Image acquisition was conducted using retrospective ECG-gated continuous scanning. The tube voltage was set at 120 kVp. Automatic exposure control (CT-AEC) was applied with a target standard deviation of 24. The gantry rotation time was 0.275 s, yielding a temporal resolution of 137.5 ms. Half reconstruction was used for image reconstruction.

An iodinated contrast agent was intravenously administered at an injection rate of 24.5 mgI/kg/s. Using bolus tracking, scanning was initiated 4 s after the attenuation in a region of interest placed in the ascending aorta reached 200 Hounsfield units (HU).

### 2.3. Image Reconstruction and Analysis

For diastolic assessment, image reconstruction was performed at 10 ms intervals to generate multiple cardiac phase images within each cardiac cycle. Because the mid-diastolic rest period is short and predictive formulas are defined in milliseconds, high temporal resolution reconstruction was adopted.

For systolic analysis, phase images were reconstructed by dividing the RR interval into 20 equal segments (RR/20) to account for heart rate dependency.

All reconstructed images were analyzed on a dedicated workstation (Ziostation REVORAS; version 5.2.2.0 Ziosoft Inc., Tokyo, Japan).

Image analysis was independently performed by three radiologic technologists. Reader 1 had 10 years of experience, whereas readers 2 and 3 each had 3 years of experience.

Optimal systolic phase assessment was conducted by reader 1 and reader 2, whereas optimal diastolic phase assessment was performed by reader 1 and reader 3.

### 2.4. Assessment of the Diastolic Coronary Rest Period

**Figure 1:**
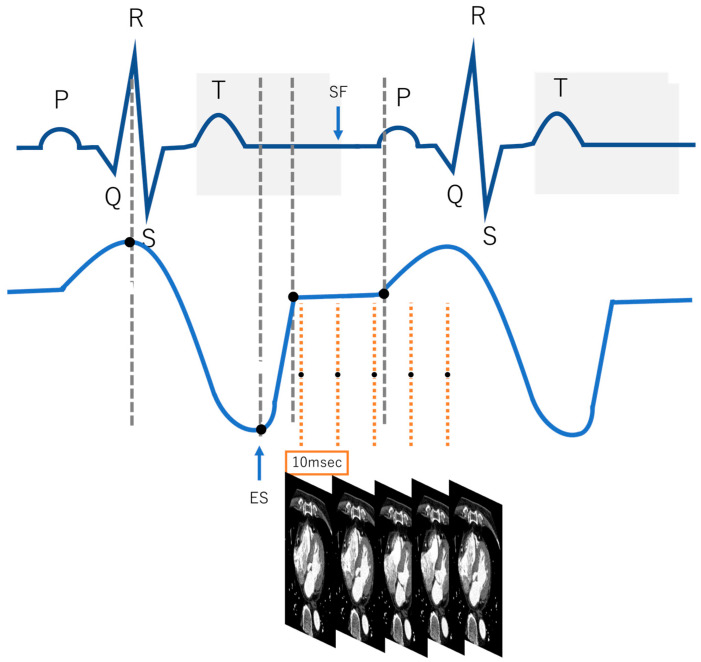
Schematic illustration of cardiac phase analysis for determination of the stationary phase (SF) in coronary CT angiography. Electrocardiographic waveform and the corresponding left ventricular volume curve were used for cardiac phase analysis. After the R-wave, the cardiac cycle was reconstructed at 10-ms intervals. For each reconstructed phase, coronary CT images were evaluated to determine the period with minimal coronary motion, defined as the stationary phase (SF). The optimal phase was selected from multiple candidate phases reconstructed throughout the cardiac cycle. Gray dashed lines indicate the reference cardiac phases used for temporal assessment. Orange dashed lines represent images reconstructed at 10-ms intervals. The blue arrows indicate the beginning and end of the stationary phase. White diamond markers represent candidate phases evaluated for coronary motion.

For the diastolic phase analysis, the slow-filling (SF) period at the proximal right coronary artery was evaluated (Figure 1). Following previous reports [3,4], a consistent cross-sectional plane including the branch with the most prominent motion was selected, and the duration of the phase with visually confirmed motion cessation was measured. The measured duration was then corrected by adding the temporal resolution of 137.5 ms to calculate the actual SF (ms). The slow-filling (SF) period was defined as the coronary diastolic rest period. The measured SF was compared with predicted values using previously reported formulas: the conventional formula and a QRS-corrected formula [6]. RR represents the RR interval recorded during CCTA acquisition, whereas PQ and QRS were obtained from standard 12-lead electrocardiography performed within 1 month of the CT examination. PQ represents the atrioventricular conduction time, and QRS represents ventricular depolarization duration.

- Conventional formula (Predicted SF):

SF = −362 + 0.742 × (RR − PQ)

- QRS-corrected formula (Corrected SF):

SF = −362 + 0.742 × {RR − PQ − (QRS − 100)}

The conventional predictive model estimates the diastolic coronary artery rest period based on RR and PQ intervals, whereas the QRS-corrected model incorporates ventricular conduction delay represented by QRS duration. This correction was introduced to compensate for the systematic prediction bias observed in patients with CLBBB by incorporating QRS duration as a practical correction term. To quantitatively evaluate the validity of SF prediction in CLBBB patients, the following regression models were constructed:

1. Simple regression model (CLBBB only):
  A simple linear regression was performed in CLBBB patients with SF as the dependent variable and RR − PQ as the independent variable:
  SF = β0 + β1 × (RR − PQ)

2. Multivariate regression model:
  A multivariate regression analysis was performed with SF as the dependent variable and both RR − PQ and QRS as independent variables:
  SF = β0 + β1 × (RR − PQ) + β2 × QRS

For each model, prediction performance was assessed using the root mean square error (RMSE) and Akaike Information Criterion (AIC). Paired *t*-tests were performed to compare the predicted SF values with the measured SF values, and the regression coefficients and their statistical significance (*p*-values) were also evaluated.

This analysis allowed us to assess whether the conventional formula is valid in CLBBB patients and to evaluate the improvement in prediction accuracy with QRS correction or regression modeling.

### 2.5. Assessment of the Optimal Systolic Phase

For systolic analysis, the right coronary artery (RCA), left anterior descending artery (LAD), and left circumflex artery (LCX) were visually evaluated throughout the course of each vessel. Phase images reconstructed at 20 equally divided cardiac phases of the RR interval (RR/20) were visually assessed for each coronary artery. The most motion-free phase throughout the course of each vessel was selected within the cardiac cycle. The selected phase was converted into absolute time (ms) and recorded. For intergroup comparison, the selected phase was further normalized by dividing by the RR interval to obtain the RR-normalized phase.

### 2.6. Statistical Analysis

Statistical analyses were performed using R (version 4.5.2; R Foundation for Statistical Computing, Vienna, Austria).

The normality of continuous variables was assessed using the Shapiro–Wilk test. In addition, given the relatively small sample size and unequal group distributions, we prespecified the use of nonparametric methods regardless of normality test results to ensure robustness of the statistical comparisons. Therefore, all group comparisons were performed using rank-based nonparametric tests. Continuous variables are presented as mean ± standard deviation and median for descriptive purposes; however, inferential statistics were conducted using nonparametric approaches. This point has been clarified in the revised manuscript. Comparisons among the three groups were performed using the Kruskal–Wallis test. When a significant difference was identified, post hoc pairwise comparisons were performed using Dunn’s test with Bonferroni correction. To evaluate the independent effects of bundle branch block, multiple linear regression analysis was performed. Regression coefficients, 95% confidence intervals, and p-values are reported. Model fit was assessed using adjusted R^2^, and multicollinearity was evaluated using variance inflation factors (VIF). Given the limited sample size in the CLBBB subgroup, regression results were interpreted as exploratory. No internal validation procedures (e.g., cross-validation or bootstrapping) or external validation were performed.

## 3. Results

### 3.1. Patient Characteristics

A total of 94 patients were included (sinus rhythm, *n* = 40; CRBBB, *n* = 36; CLBBB, *n* = 18). QRS duration was significantly prolonged in both the CRBBB and CLBBB groups compared with the sinus rhythm group (*p* < 0.001). No significant differences were observed in heart rate (HR) or RR interval among the three groups (*p* > 0.05) (Table 1).

### 3.2. Comparison of Measured and Predicted Slow-Filling Periods

**Figure 2:**
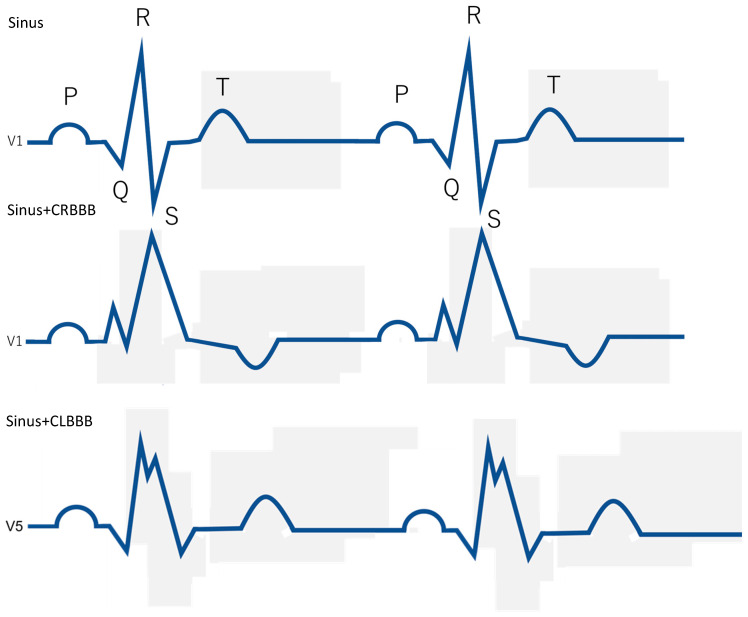
Representative coronary CT angiograms across the three groups. Representative mid-diastolic rest phase images in patients with sinus rhythm, complete right bundle branch block (CRBBB), and complete left bundle branch block (CLBBB).

**Figure 3:**
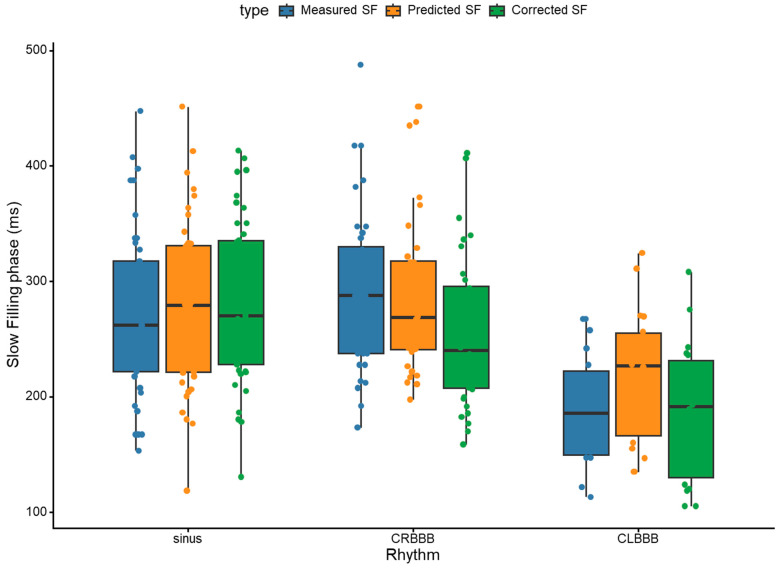
Comparison of measured, predicted, and corrected slow-filling phase durations among the three groups. Box plots showing the distribution of the slow-filling (SF) phase in patients with sinus rhythm, complete right bundle branch block (CRBBB), and complete left bundle branch block (CLBBB). Measured SF, predicted SF derived from the conventional model, and corrected SF obtained using the proposed correction model are shown for each rhythm group. Individual points represent measured data for each patient. Red circles indicate measured SF, blue circles indicate predicted SF, and green circles indicate corrected SF.

The mean values of the measured slow filling (SF) and predicted/corrected SF obtained at the proximal right coronary artery for each group are summarized in Table 2. Furthermore, representative coronary artery images and optimal stationary phases for each group are shown in Figure 2. While no significant difference was observed in the measured SF between the sinus rhythm and CRBBB groups, the SF in the CLBBB group was significantly shorter than in the other two groups (Figure 3). A detailed comparison of the predictive performance of each model in CLBBB patients is provided in Table 3.

**Figure 4:**
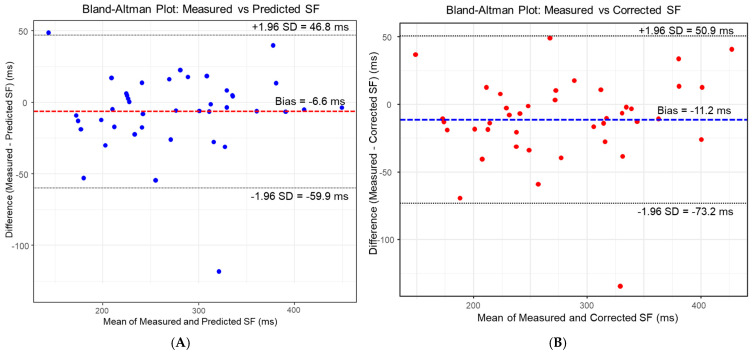
Bland–Altman analysis comparing measured stationary phase (SF) with conventional predicted SF and proposed corrected SF across all patients. Bland–Altman plots illustrating the agreement between the measured SF and the SF predicted by the conventional formula (‘SF = −362 + 0.742 × [RR − PQ]’) (**A**), and the agreement between the measured SF and the SF corrected by the proposed QRS-adjusted formula (‘SF = −362 + 0.742 × {RR − PQ − [QRS − 100]}’) (**B**) for the entire study cohort of 94 patients. In plot (**A**), patients with complete left bundle branch block (CLBBB) appear as a distinct cluster with a systematic overestimation bias of approximately +38 ms. Plot (**B**) demonstrates that applying the QRS correction successfully eliminates this bias, unifying the entire cohort around the zero-error line. The dashed line represents the mean difference (bias), and the dotted lines represent the 95% limits of agreement. In panel (**A**), blue circles represent individual patients and the red dashed line indicates the mean difference (bias). In panel (**B**), red circles represent individual patients and the blue dashed line indicates the mean difference (bias).

**Figure 5:**
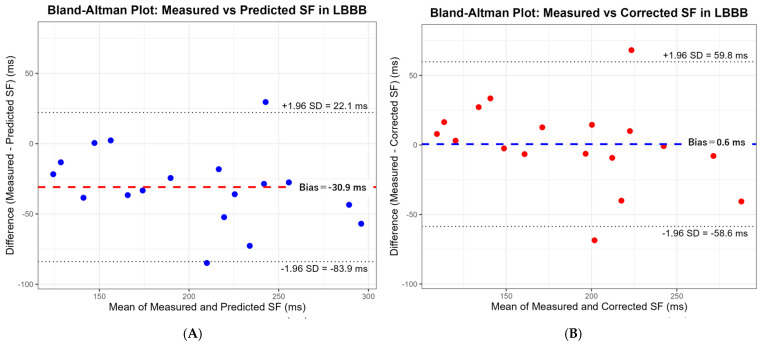
Bland–Altman analysis evaluating the efficacy of the QRS-corrected formula specifically in patients with complete left bundle branch block (CLBBB). Bland–Altman plots isolating the 18 patients in the CLBBB group to compare the agreement between measured SF and conventionally predicted SF (**A**), and measured SF and QRS-corrected SF (**B**). Plot (**A**) highlights the systematic overestimation (mean bias, +37.9 ms) observed when the conventional formula was applied to CLBBB patients. Plot (**B**) demonstrates that the proposed QRS-corrected formula substantially reduces this systematic prediction bias, decreasing the mean bias to near zero (−6.9 ms) and improving agreement with the measured SF.

**Figure 6:**
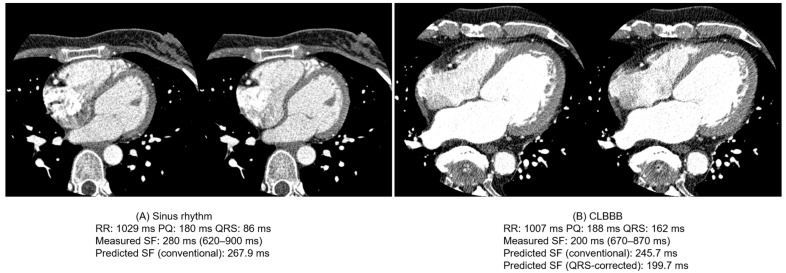
Representative CCTA Cases Demonstrating Shortened Diastolic Coronary Rest Period in CLBBB Despite Similar RR and PQ Intervals.

These results are further illustrated in Figure 4. Bland–Altman analysis in CLBBB patients is shown in Figure 5. Representative CCTA cases demonstrating the shortened diastolic coronary rest period in CLBBB are shown in Figure 6. Additional Bland–Altman analyses are provided in Figures S1 and S2. he correlation between measured and predicted SF is shown in Figures S3 and S4.

Predicted formula:

SF = −362 + 0.742 × (RR − PQ)

Application of this formula yielded an RMSE of 42.5 ms and an AIC of 160.1. The paired t-test revealed a mean difference of −37.9 ms (*p* = 2.7 × 10^−7^) between measured and predicted SF, indicating that the conventional formula systematically overestimated SF by approximately 38 ms in CLBBB patients.

Corrected formula:

SF = −362 + 0.742 × {RR – PQ − (QRS − 100)}

Using this formula, the RMSE decreased to 21.2 ms and the AIC to 159.2. The paired *t*-test showed a mean difference of −6.9 ms (*p* = 0.176), indicating no statistically significant difference from the measured SF. The predictive error was substantially improved compared with the conventional formula.

A multivariate regression analysis with SF as the dependent variable and RR − PQ and QRS as independent variables resulted in:

SF = −295.6 + 0.695 × (RR − PQ) − 0.465 × QRS

RR − PQ was statistically significant (*p* < 0.001), while QRS was not (*p* = 0.318). Residual SE was 18.51, and AIC was 160.9.

In a univariate regression analysis restricted to CLBBB patients,

SF = −368.5 + 0.704 × (RR − PQ)

Residual SE was 18.55. The regression coefficient was similar to that of the conventional formula, and although the regression coefficient was similar to that of the conventional formula, the CLBBB-specific regression model did not provide a substantial advantage over the QRS-corrected formula.

Overall, these results indicate that the fundamental relationship between RR − PQ and SF is largely preserved in CLBBB patients. However, a systematic overestimation was observed in patients with CLBBB when the conventional formula was applied. The QRS-adjusted formula substantially improved prediction accuracy compared with the conventional formula and achieved predictive performance comparable to that of the regression-based models.

As a supplementary analysis, the predictive accuracy of the conventional formula was also evaluated in the CRBBB group (Figure 1 and Figure 3). The mean difference between the measured SF (296.98 ms) and the predicted SF (288.05 ms) was minimal (approximately 9 ms), demonstrating good agreement. This confirms that the right ventricular conduction delay in CRBBB has a limited direct impact on left ventricular diastolic dynamics, and that the conventional formula retains acceptable predictive accuracy for CRBBB patients.

### 3.3. Systolic Analysis

Comparison of RR-normalized systolic quiescent phases among the three groups revealed significant differences in LAD and LCX (LAD: *p* = 0.0188, LCX: *p* = 0.0116).

Post hoc pairwise comparisons using Dunn’s test with Bonferroni correction showed a significant difference between the CLBBB and sinus rhythm groups in LAD (*p* = 0.018), and between CLBBB and the sinus rhythm group (*p* = 0.017), as well as CLBBB and CRBBB (*p* = 0.020) in LCX.

No significant differences were observed between the CRBBB and sinus groups in any vessel. No significant difference was detected among the three groups in RCA (*p* = 0.831).

### 3.4. Multiple Linear Regression Analysis (QRS + HR Model)

**Figure 7:**
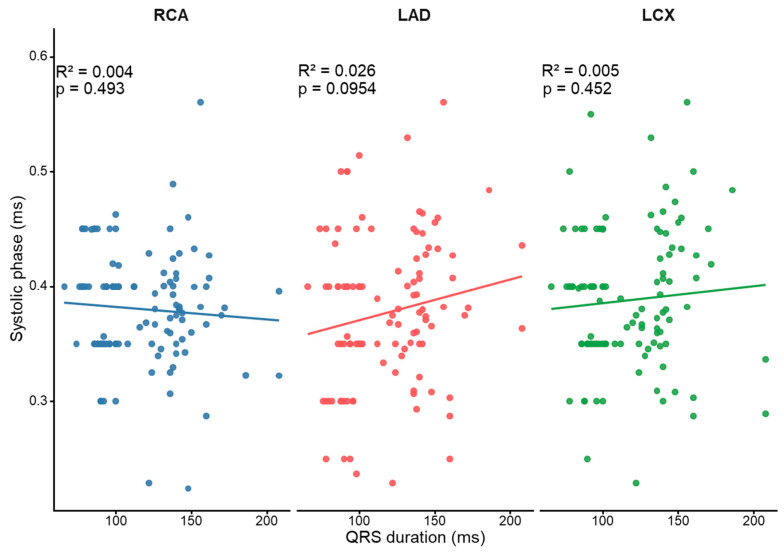
Relationship between QRS duration and RR-normalized systolic stationary phase in the three major coronary arteries. Scatter plots showing the relationship between QRS duration and the RR-normalized systolic stationary phase in the RCA, LAD, and LCX. A positive trend was observed particularly in the LCX, whereas the association was less apparent in the RCA and LAD.

LCX–RCA phase difference showed significant associations with both QRS duration (*p* = 0.005) and heart rate (*p* = 0.047), with an adjusted R^2^ of 0.41. In this model, QRS duration and HR were both significant predictors, indicating an independent association with inter-vessel phase difference. In contrast, no significant associations were observed for LAD–RCA phase difference (QRS: *p* = 0.112; HR: *p* = 0.516), and the model was not statistically significant (adjusted R^2^ = 0.06). These relationships are illustrated in Figure 7. The relationship between prediction error and heart rate in the CLBBB group is shown in Figure S5.

### 3.5. Interobserver Agreement

Interobserver agreement for diastolic SF demonstrated good agreement:

ICC(2,1) = 0.871 (95% CI, 0.706–0.947; *p* < 0.001).

Interobserver agreement for optimal systolic phase demonstrated excellent agreement:

ICC(2,1) = 0.948 (95% CI, 0.916–0.968; *p* < 0.001).

## 4. Discussion

In this study, we quantitatively compared the cardiac phase characteristics of coronary CT among three groups: sinus rhythm, CRBBB, and CLBBB, and investigated the effects of bundle branch block on diastolic and systolic resting phases. The results demonstrated that the slow-filling (SF) duration during diastole was significantly shorter in the CLBBB group, and the conventional prediction formula significantly overestimated SF compared with the measured values. In contrast, applying a QRS-adjusted formula eliminated the significant difference between predicted and measured SF and substantially improved predictive accuracy.

### 4.1. Effect of CLBBB on Diastolic Resting Duration

CLBBB is known to cause delayed activation of the left ventricular free wall, resulting in electrical and mechanical dyssynchrony within the left ventricle [13,14,15]. This dyssynchronous activation leads to regional differences in contraction timing, including delayed septal activation and late lateral wall contraction, which may subsequently influence both systolic performance and early diastolic relaxation. In the present study, SF was shortened in the CLBBB group, suggesting that ventricular electrical–mechanical interaction may influence diastolic resting duration. In contrast, no significant difference was observed between the CRBBB and sinus rhythm groups, indicating that delayed right ventricular activation has a limited effect on left ventricular diastolic dynamics. However, CLBBB represents a heterogeneous electrophysiological condition. The degree and pattern of mechanical dyssynchrony may vary considerably among individuals, depending on underlying myocardial substrate, ventricular remodeling, and functional status. Therefore, CLBBB should be considered a clinical surrogate marker rather than a uniform pathophysiological entity. To further characterize this heterogeneity, available echocardiographic parameters in patients with CLBBB are presented in Table 4. Although echocardiographic data were available for only 14 of 18 patients and were not acquired according to a standardized protocol relative to CCTA, the findings demonstrate variability in left ventricular systolic function, chamber size, and diastolic parameters within the CLBBB cohort. These observations support the concept that CLBBB encompasses a broad spectrum of underlying cardiac physiology. Importantly, the present study did not include direct assessment of mechanical dyssynchrony using advanced echocardiographic parameters such as septal flash, apical rocking, tissue Doppler-derived mechanical delays, or strain imaging. Accordingly, the observed shortening of SF is interpreted as a global consequence of altered electromechanical activation rather than a direct quantification of dyssynchrony severity. Taken together, these findings suggest that the observed changes in diastolic resting duration are more likely attributable to heterogeneous electromechanical alterations associated with CLBBB, rather than QRS prolongation alone.

### 4.2. Limitations of the Conventional Formula and the Significance of QRS Correction

The conventional SF prediction formula was originally developed in patients with normal intraventricular conduction and estimates the duration of the diastolic coronary rest period using the RR interval and PQ duration [11]. In the present study, application of this formula to patients with CLBBB resulted in a systematic overestimation of SF by approximately 38 ms. This finding indicates that the conventional model does not fully account for the altered electromechanical activation pattern associated with CLBBB. One possible explanation is that delayed ventricular activation prolongs the interval between electrical depolarization and completion of mechanical contraction. As a result, part of the interval represented by RR − PQ may no longer contribute to the effective diastolic resting period in the same manner as in patients with normal conduction. Consequently, the conventional formula may overestimate the duration of coronary quiescence in CLBBB. When the QRS-corrected formula was applied, prediction error was substantially reduced and the difference between predicted and measured SF was no longer statistically significant. These findings suggest that incorporating QRS prolongation can compensate for the systematic bias observed with the conventional formula. Importantly, however, multivariate regression analysis demonstrated that RR − PQ remained a significant predictor of SF, whereas QRS duration was not retained as an independent predictor. This observation indicates that the fundamental relationship between RR − PQ and SF is largely preserved even in the presence of abnormal ventricular conduction. The lack of an independent association between QRS duration and SF suggests that QRS prolongation should not be interpreted as a direct determinant of diastolic resting duration. Rather, QRS duration may function as a practical surrogate marker reflecting the broader electromechanical alterations associated with CLBBB. Therefore, the improved performance of the QRS-corrected formula should be viewed as a correction for systematic prediction bias rather than evidence of a direct causal relationship between QRS duration and coronary motion. Although the regression-based models demonstrated slightly lower prediction errors than the QRS-corrected formula, these models require fitting within a specific CLBBB dataset and may be more susceptible to sample-dependent variation. In contrast, the proposed QRS-corrected formula preserves the simple structure of the original predictive model while substantially improving agreement with measured SF. From a practical perspective, this approach may be more suitable for routine clinical application because it can be implemented using standard electrocardiographic parameters without the need for additional model development. Taken together, these findings suggest that the electromechanical alterations associated with CLBBB may contribute to shortening of the diastolic resting period. However, because direct measures of mechanical dyssynchrony were not available in the present study, the precise physiological mechanisms underlying this phenomenon remain incompletely understood and warrant further investigation.

### 4.3. Systolic Phase Differences and Vessel-Specific Findings

During systole, significant differences were observed in the LAD and LCX, whereas no significant difference was found in the RCA. In particular, the LCX–RCA phase difference was independently associated with QRS duration and heart rate, suggesting that conduction abnormalities associated with CLBBB may influence the temporal distribution of coronary artery motion. The LCX runs along the left ventricular free wall and may therefore be more susceptible to delayed lateral wall contraction in CLBBB [14,15].

### 4.4. Reproducibility

Interobserver agreement for diastolic SF and systolic optimal phase was good to excellent, with ICC(2,1) values of 0.871 and 0.948, respectively. In particular, the systolic phase showed excellent agreement, confirming the high reproducibility of visual phase selection. Although the agreement for diastolic SF was slightly lower than that for systolic measurements, it remained within an acceptable clinical range.

### 4.5. Clinical Implications

The selection of the optimal cardiac phase in coronary CT has a substantial impact on image quality. Mid-diastolic resting phases are generally used for image reconstruction; however, in patients with CLBBB, applying the conventional prediction formula may lead to an overestimation of the optimal diastolic phase. The present findings suggest that incorporating QRS correction may allow a more accurate estimation of diastolic resting duration in CLBBB patients. From a practical perspective, the proposed QRS-corrected formula can be easily incorporated into routine CCTA workflow because it requires only standard electrocardiographic parameters available before scanning. More accurate estimation of the diastolic resting phase may facilitate more appropriate phase selection in patients with CLBBB.

### 4.6. Study Limitations

This study has several limitations. First, the number of patients with CLBBB was relatively small. However, this reflects the relatively low prevalence of CLBBB among patients undergoing CCTA in clinical practice. Despite the limited sample size, statistically significant differences were detected in both diastolic and systolic phase characteristics.

Second, this was a single-center retrospective study. Mechanical dyssynchrony was not directly evaluated using functional imaging modalities such as echocardiography. In addition, the QRS-adjusted formula was validated only in CLBBB patients, and its applicability to other conduction abnormalities or patients with high heart rates requires further investigation.

Third, determination of the slow-filling phase was based on visual assessment of coronary motion and therefore may be partially subjective. Although interobserver agreement was good, objective quantitative methods for motion analysis may provide a more robust assessment of coronary resting phases in future studies.

Fourth, the regression analyses were performed in a relatively small CLBBB cohort, which may limit model stability and increase the risk of overfitting. No internal validation procedures, such as bootstrapping or cross-validation, were performed. Therefore, the regression results and the QRS-adjusted formula should be interpreted as exploratory findings that warrant confirmation in larger independent cohorts. In particular, the lack of direct assessment of mechanical dyssynchrony (e.g., septal flash, apical rocking, or strain-based delay) is an important limitation, and future studies should integrate advanced echocardiographic or imaging-based markers. This limitation further supports the concept that CLBBB represents a heterogeneous physiological substrate rather than a uniform entity.

## 5. Conclusions

In conclusion, CLBBB was associated with a shorter diastolic slow-filling duration, and the conventional prediction formula tended to overestimate SF in these patients. Application of a simple QRS-adjusted correction improved agreement between predicted and measured SF values. The relationship between RR − PQ and SF appeared to be largely preserved despite abnormal intraventricular conduction, suggesting that a simple correction approach may be useful in selected patients for estimating diastolic resting duration in CLBBB. Although the proposed strategy may have potential clinical utility for cardiac phase selection in coronary CT angiography, it should be interpreted as an exploratory finding. Further validation in larger and independent cohorts is required before clinical application.

## Figures and Tables

**Table 1:** Patient Characteristics.

	Sinus (*n* = 40)	Sinus + CRBBB (*n* = 36)	Sinus + CLBBB (*n* = 18)
Age (years)	67.25 ± 12.2	69.84 ± 13.5	70.31 ± 15.1
	71.5 (62.2–75.9)	71.5 (63.0–81.1)	77.0 (67.5–79.2)
Sex	Men 23/Female 17	Men 35/Female 5	Men 6/Female 12
Heart rate (bpm)	58.93 ± 5.9	68.7 ± 14.2	67.20 ± 12.6
	58 (54.0–63.1)	69 (59.2–80.2)	63.9 (60.7–69.6)
RR interval (ms)	1028.0 ± 100.7	1037.6 ± 95.2	929.8 ± 213.8
	1027.0 (950.5–1101.6)	1012.0 (967.2–1076.8)	938.5 (860.4–994.1)
PQ interval (ms)	157.8 ± 21.5	162.1 ± 25.6	164.3 ± 21.6
	158.0 (144.2–172.5)	157.0 (142.6–182.3)	167.0 (158.2–182.3)
QRS duration (ms)	94.5 ± 16.3	141.4 ± 22.7	141.8 ± 16.0
	92.0 (86.9–99.7)	138.0 (132.3–144.8)	143.0 (136.3–149.5)
β-blocker use(%)	28/40 (70%)	20/36 (55%)	13/18 (72%)

Data are presented as mean ± standard deviation for continuous variables or as number (percentage) for categorical variables. CRBBB, complete right bundle branch block; CLBBB, complete left bundle branch block.

**Table 2:** Comparison of measured and predicted slow-filling (SF) durations across the three groups.

	Sinus (*n* = 40)	Sinus + CRBBB (*n* = 36)	Sinus + CLBBB (n = 18)
Measured SF (ms)	270.7 ± 72.8	297.0 ± 67.7	215.6 ± 47.5
	261.9 (220.1–315.5)	287.5 (238.8–330.6)	185.5 (153.0–218.4)
SF/RR	0.26 ± 0.05	0.28 ± 0.06	0.21 ± 0.06
	0.25 (0.21–0.29)	0.27 (0.24–0.30)	0.20 (0.17–0.23)
Predicted SF (ms)	277.3 ± 71.5	288.1 ± 69.6	246.4 ± 55.2
	278.1 (222.8–330.1)	268.3 (241.6–318.4)	226.8 (172.3–254.4)
Corrected SF (ms)	281.8 ± 97.1	257.3 ± 67.1	215.0 ± 56.8
	270.2 (229.4–335.5)	239.8 (207.4–296.1)	218.1 (137.2–227.7)

Data are expressed as mean ± standard deviation. SF, slow filling.

**Table 3:** Comparison of conventional, regression, and QRS-corrected predictive models for diastolic stationary phase (SF) in patients with complete left bundle branch block (CLBBB).

Predictive Models	Formula	RMSE (ms)	AIC	Mean Error (ms)
Conventional formula	SF = −362 + 0.742 × (RR − PQ)	42.5	160.1	−37.9
Simple regression model	SF = −368.5 + 0.704 × (RR − PQ)	18.55	-	-
Multiple regression model	SF = −295.6 + 0.695 × (RR − PQ) − 0.465 × QRS	18.51	160.9	-
QRS-corrected formula	SF = −362 + 0.742 × {RR − PQ − (QRS − 100)}	21.2	159.2	−6.9

Conventional formula, simple regression, and multivariate regression models were evaluated exclusively in the CLBBB group and compared against the proposed QRS-corrected formula. RMSE, root mean square error; AIC, Akaike information criterion.

**Table 4:** Echocardiographic characteristics in patients with CLBBB.

	LVEF (%)	LVEDV (mL)	LVESV (mL)	LVH (*n*)	LV Dilatation (*n*)	E/A	DcT (ms)	E/E’	E’ (cm/s)
Median	56.5 (47.25–64.75)	90.5 (53–124)	40.5 (22.3–62.3)	2/14	2/14	0.7 (0.6–0.9)	177.5 (138.3–197.8)	11 (10–13.5)	6 (4–7)

Data are presented as median (interquartile range). Echocardiographic data were available in 14 of 18 patients with CLBBB. LVEF was measured using the modified biplane Simpson method.

## Data Availability

The data presented in this study are not publicly available due to privacy and ethical restrictions. Data may be available from the corresponding author upon reasonable request and with permission from the institutional ethics committee.

## Supplementary Materials

The following supporting information can be downloaded at https://www.mdpi.com/article/10.3390/jcdd13060285/s1, Figure S1. Bland–Altman analysis between measured and predicted SF in the CRBBB group; Bland–Altman plot showing the agreement between the measured stationary phase (SF) and the predicted SF in the CRBBB group. Figure S2. Bland–Altman analysis between measured and predicted SF in the CLBBB group. Bland–Altman plot showing the agreement between the measured stationary phase (SF) and the predicted SF in the CLBBB group; Figure S3. Correlation between measured and predicted SF in the CRBBB group. Scatter plot showing the relationship between the measured stationary phase (SF) and the predicted SF in the CRBBB group. The solid line represents the linear regression line, and the dashed line represents the line of identity (y = x); Figure S4. Correlation between measured and predicted SF in the CLBBB group. Scatter plot showing the relationship between the measured stationary phase (SF) and the predicted SF in the CLBBB group. The solid line represents the linear regression line, and the dashed line represents the line of identity (y = x). Figure S5. Relationship between prediction error and heart rate in the CLBBB group. Scatter plot showing the relationship between prediction error (measured SF − predicted SF) and heart rate in the CLBBB group. Linear regression analysis showed no significant association between prediction error and heart rate.

## Abbreviations

CCTA	coronary computed tomography angiography
CT	computed tomography
SF	slow filling
CRBBB	complete right bundle branch block
CLBBB	complete left bundle branch block
RCA	right coronary artery
LAD	left anterior descending artery
LCX	left circumflex artery
HR	heart rate
RMSE	root mean square error
AIC	Akaike information criterion
ICC	intraclass correlation coefficient
