# Supplementary material for: QRS-Corrected Prediction of the Diastolic Rest Period for Coronary CT Angiography in Patients with Complete Left Bundle Branch Block
Source: J Cardiovasc Dev Dis. 2026 Jun 22;13(6):285. doi: 10.3390/jcdd13060285 (PMC13302584; doi:10.3390/jcdd13060285)
Supplement: Supplementary file 1 [file jcdd-13-00285-s001.zip › jcdd-4261458-supplementary.pdf]

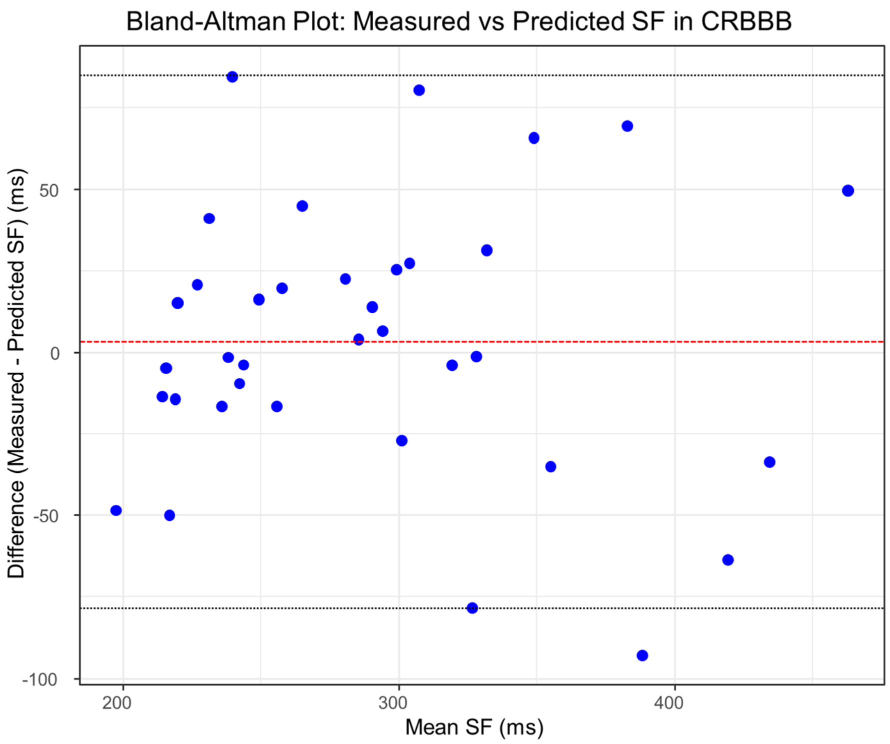

Figure S1. Bland–Altman analysis between measured and predicted SF in the CRBBB group. Bland–Altman plot showing the agreement between the measured stationary phase (SF) and the predicted SF in the CRBBB group.

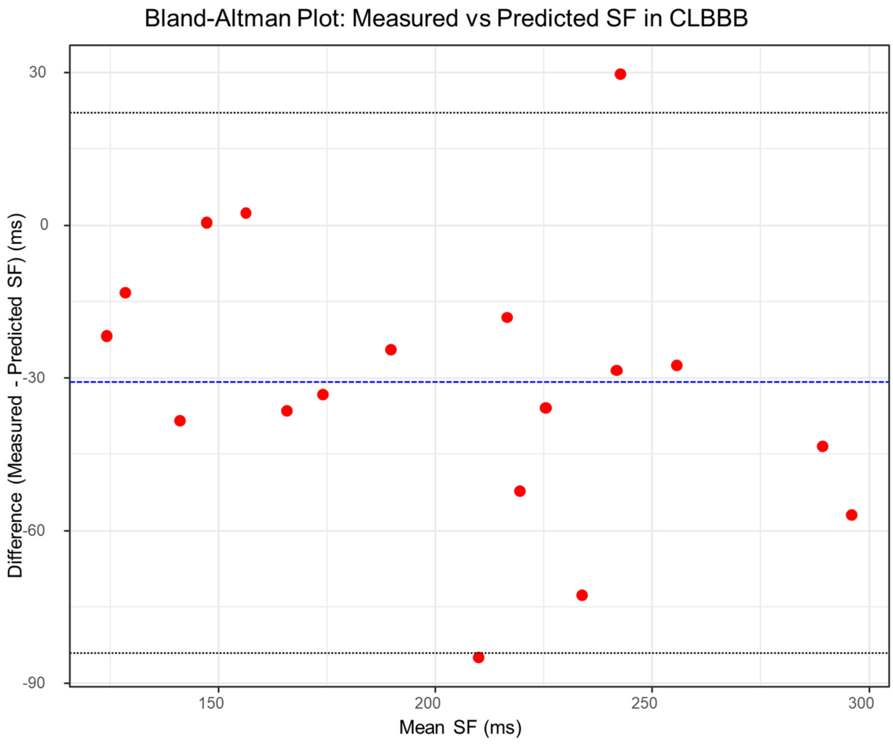

Figure S2. Bland–Altman analysis between measured and predicted SF in the CLBBB group. Bland–Altman plot showing the agreement between the measured stationary phase (SF) and the predicted SF in the CLBBB group.

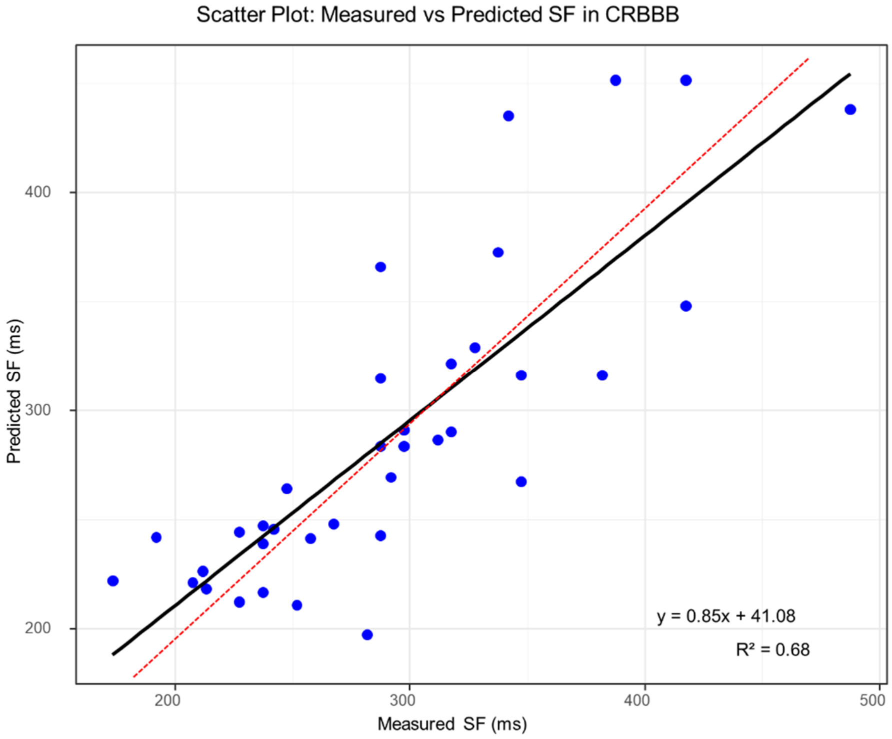

Figure S3. Correlation between measured and predicted SF in the CRBBB group. Scatter plot showing the relationship between the measured stationary phase (SF) and the predicted SF in the CRBBB group. The solid line represents the linear regression line, and the dashed line represents the line of identity (y = x).

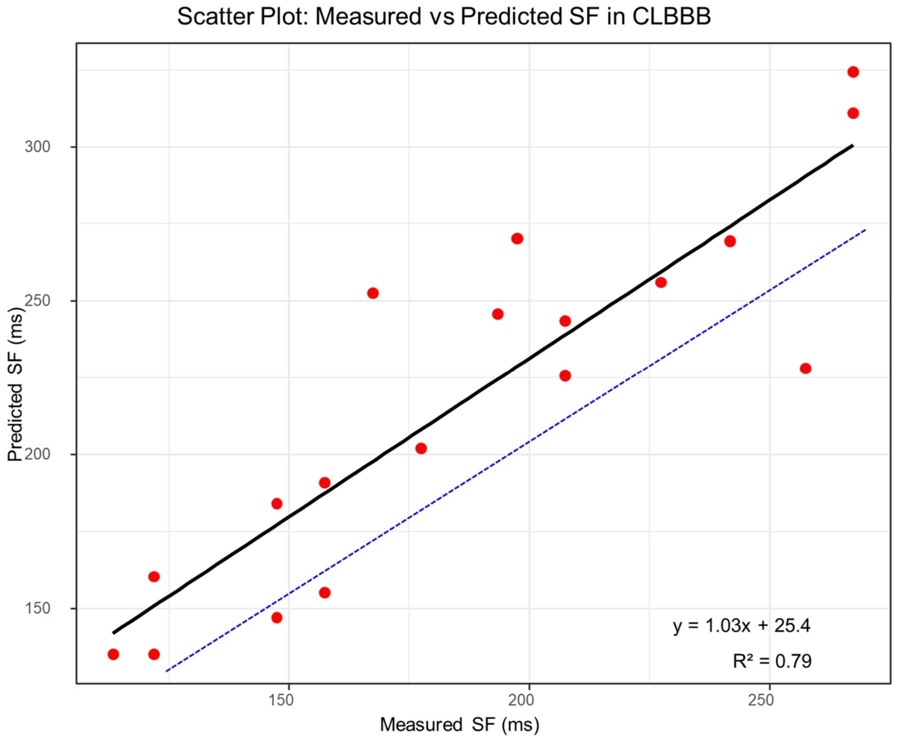

Figure S4. Correlation between measured and predicted SF in the CLBBB group. Scatter plot showing the relationship between the measured stationary phase (SF) and the predicted SF in the CLBBB group. The solid line represents the linear regression line, and the dashed line represents the line of identity (y = x).

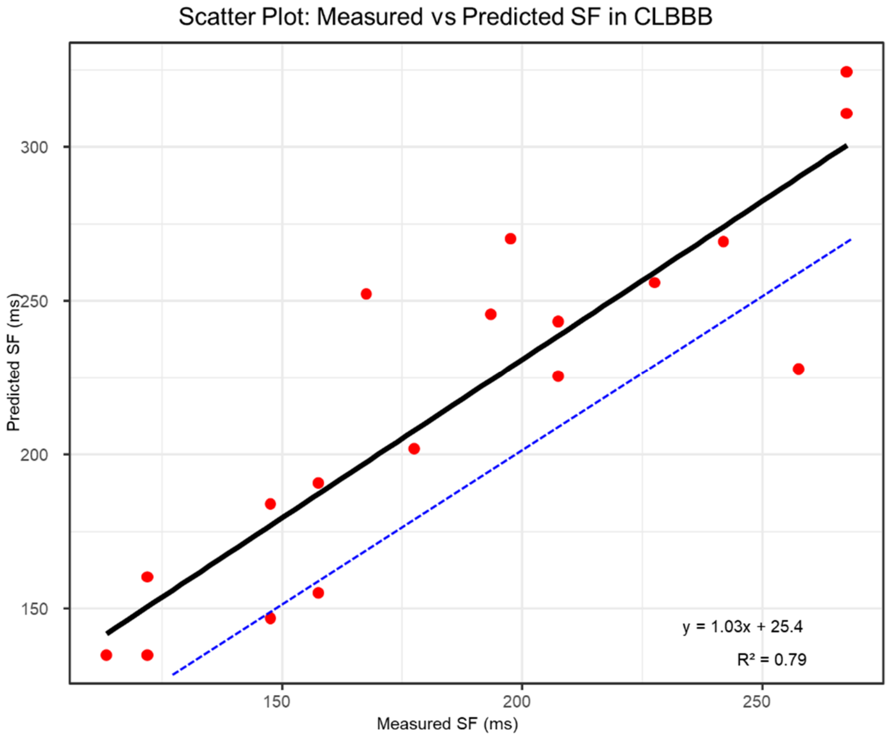

Figure S5. Relationship between prediction error and heart rate in the CLBBB group. Scatter plot showing the relationship between prediction error (measured SF − predicted SF) and heart rate in the CLBBB group. Linear regression analysis showed no significant association between prediction error and heart rate.
